# DDT and Breast Cancer Trends

**DOI:** 10.1289/ehp.11562

**Published:** 2008-09

**Authors:** Robert E. Tarone

**Affiliations:** International Epidemiology Institute Rockville, Maryland E-mail: bob@intepi.org

Cohn et al. (2008) suggested that birth cohort trends in breast cancer rates for women under 50 years of age are consistent with declining use of DDT (dichloro-diphenyltrichloroethane) after 1959. They cited Weiss (2007) in claiming that increased detection and treatment of *in situ* breast cancer must be considered when interpreting recent trends in breast cancer mortality rates in young women. The remarks of Weiss (2007) relate to women 40–49 years of age, and earlier detection and improved treatment of breast cancer has had a marked impact on breast cancer mortality rates in these women since 1990 (Berry et al. 2005; Chu et al. 1996). The birth cohort trends relevant to examining the possible impact of childhood DDT exposure on U.S. breast cancer rates, however, were firmly established well before 1990 in women < 40 years of age (Tarone 2007).

Cohn et al. (2007) reported a large increase in breast cancer risk estimates for *p,p′*-DDT [1,1,1-trichloro-2,2-bis(*p*-chlorophenyl)ethane] exposure with successive birth cohorts after 1930. Their reported odds ratio estimates by period of birth for the highest tertile of *p,p′*-DDT exposure were 0.6 for women born in 1931 or earlier (i.e., ≥ 14 years of age in 1945), 3.9 for women born in 1932–1937 (i.e., 8–13 years of age in 1945), 9.6 for women born in 1938–1941 (i.e., 4–7 years of age in 1945), and 11.5 for women born in 1942 or later (i.e., < 4 years of age in 1945) [Table 4, Cohn et al. (2007)]. In contrast, I have found no evidence of increasing breast cancer rates among young U.S. women born between 1930 and 1945 (Tarone 2007). I quantified trends in breast cancer mortality rates for U.S. white women 20–39 years of age (by 5-year age group) born during 1930–1945 using linear regression analyses with the logarithm of the age-specific rate as the dependent variable and year of birth as the independent variable (with two-sided *p*-values) [Surveillance, Epidemiology, and End Results (SEER) 2006; Tarone 2007]. The slope estimates did not differ significantly from zero for women in the three youngest age groups (*p* > 0.25), and there was a marginally significant decrease in rates for women 35–39 years of age (*p* = 0.04). Thus the trends in breast cancer mortality rates among women born in 1930–1945 are not consistent with the sharply increasing trend in odds ratios for childhood DDT exposure by birth period reported by Cohn et al. (2007). The most recent mortality rate contributing to the reported regression analyses (corresponding to women in the 35- to 39-year age group born in 1945) was for 1983, well before improvements in detection and treatment would have had any impact on breast cancer mortality rates.

Women born after 1945 were exposed to DDT for each of the first 13 years of life (and all years thereafter). In addition, DDT exposure increased from 1945 through 1959, when DDT use peaked (with dietary exposure peaking in 1965) (Wolff et al. 2005). If DDT exposure early in life markedly increases breast cancer risk, then some evidence of the increasing DDT use after 1945 might be expected in breast cancer mortality rate trends for young women born from 1946 through 1959 (Tarone 2007). Breast cancer mortality rates decreased significantly among women 20–24 years of age (*p* = 0.009) and 25–29 years of age (*p* = 0.0002) born between 1946 and 1959 (SEER 2006; Tarone 2007). The most recent rate contributing to these regression analyses was for 1987 (corresponding to women in the 25-to 29-year age group born in 1959). Breast cancer mortality rates decreased even more markedly (*p* < 0.0001) for women in the 30- to 34-year and 35- to 39-year age groups born from 1946 through 1959; some of the recent rates in these latter age groups were almost certainly affected by improved breast cancer detection and treatment, although decreasing trends were apparent in both age groups for rates well before 1990 (Tarone 2007). Thus, U.S. breast cancer mortality rates in women between the ages of 20 and 39 who were born between 1930 and 1959 show no evidence of an increase in breast cancer risk associated with their marked increase in DDT exposure during childhood.

The observed birth cohort trends in breast cancer rates do not refute a possible association between childhood DDT exposure and breast cancer risk, and contrary to the implication of Cohn et al. (2008), no such claim was made in my earlier letter (Tarone 2008). The regression analyses reported above suffer the weaknesses of all ecologic analyses, and in fact, the decreasing birth cohort risk of breast cancer in baby boomers has been observed in spite of trends in established risk factors (e.g., parity, age at first birth, and oral contraceptive use) that would predict increasing breast cancer rates among U.S. women born after 1945. If, as suggested by Cohn et al. (2007), the public health significance of DDT exposure early in life is large, then this would provide additional evidence that the factor or factors responsible for the paradoxical decrease in birth cohort risk of breast cancer observed among U.S. baby boomers must have a very powerful impact on breast cancer etiology, large enough to turn an expected increasing trend in breast cancer rates among baby boomers into a decreasing trend.

## Editor’s note

In accordance with journal policy, Cohn et al. were asked whether they wanted to respond to this letter, but they chose not to do so.
